# The importance of digitized biocollections as a source of trait data and a new VertNet resource

**DOI:** 10.1093/database/baw158

**Published:** 2016-12-26

**Authors:** Robert P. Guralnick, Paula F. Zermoglio, John Wieczorek, Raphael LaFrance, David Bloom, Laura Russell

**Affiliations:** 1 University of Florida Museum of Natural History University of Florida at Gainesville, Gainesville, FL, USA; 2 Departamento de Ecología, Genética y Evolución, Instituto IEGEBA (CONICET-UBA), Facultad de Ciencias Exactas y Naturales, Universidad de Buenos Aires, Buenos Aires, Argentina; 3 Institut de Recherche sur la Biologie de l’Insecte, UMR 7261 CNRS, Université François Rabelais, Tours, France; 4 Museum of Vertebrate Zoology University of California, Berkeley, CA, USA; 5 Biodiversity Institute University of Kansas, Lawrence, KS, USA

## Abstract

For vast areas of the globe and large parts of the tree of life, data needed to inform trait diversity is incomplete. Such trait data, when fully assembled, however, form the link between the evolutionary history of organisms, their assembly into communities, and the nature and functioning of ecosystems. Recent efforts to close data gaps have focused on collating trait-by-species databases, which only provide species-level, aggregated value ranges for traits of interest and often lack the direct observations on which those ranges are based. Perhaps under-appreciated is that digitized biocollection records collectively contain a vast trove of trait data measured directly from individuals, but this content remains hidden and highly heterogeneous, impeding discoverability and use. We developed and deployed a suite of openly accessible software tools in order to collate a full set of trait descriptions and extract two key traits, body length and mass, from >18 million specimen records in VertNet, a global biodiversity data publisher and aggregator. We tested success rate of these tools against hand-checked validation data sets and characterized quality and quantity. A post-processing toolkit was developed to standardize and harmonize data sets, and to integrate this improved content into VertNet for broadest reuse. The result of this work was to add more than 1.5 million harmonized measurements on vertebrate body mass and length directly to specimen records. Rates of false positives and negatives for extracted data were extremely low. We also created new tools for filtering, querying, and assembling this research-ready vertebrate trait content for view and download. Our work has yielded a novel database and platform for harmonized trait content that will grow as tools introduced here become part of publication workflows. We close by noting how this effort extends to new communities already developing similar digitized content.

**Database URL**: http://portal.vertnet.org/search?advanced=1

## Introduction

A trait constitutes any measurable or observable morphological, structural, behavioral, physiological, or phenological characteristic of an organism. Traits are expressed as phenotypes, with respect to which key aspects of organismal fitness and ecological roles in communities are defined. Comprehensive taxonomic and geographic views of trait diversity enable ecological and evolutionary characterizations of populations, species and communities, and constitute a powerful tool to understand their temporal (1), spatial (2), and community dynamics (3). Given their central importance in biology, trait data have been captured and reported broadly for centuries, but have only recently begun to be re-assembled into global trait databases (4–6). Despite these efforts, digitally accessible trait data remain grossly incomplete and non-representative taxonomically, geographically, environmentally, temporally and functionally. Concerted efforts to close gaps, even when considering the most impressive initiatives to assemble and aggregate global scale data sets, such as the TRY plant function database (www.try-db.org, 7), are far from being complete. These efforts present spatial undersampling of at least an order of magnitude for most areas, especially in the tropics (8).

The predominant digitally accessible resources for vertebrate traits are a repackaging of information from primary or secondary literature (6, 9, 10), where trait mean values or ranges are associated with species names. These trait-by-species compilations, while valuable, provide few ways to assess quality (e.g. they lack pointers to the original observations, sample sizes and variances) and largely ignore variation among individuals. However, this missing information could provide fundamental insight into biological mechanisms at multiple spatial and temporal scales (11). Relating trait variation to underlying genetic or ecological variation is critical, e.g. to understand the genetic basis of adaptive change or the impact of climatic or habitat change (12). Maintaining trait data associations to individual organisms offers a number of advantages, because it allows: (i) aggregation of traits and production of distributions of trait variation within species, including new modeling techniques for trait evolution that utilize this information (13); (ii) detection of outlying trait values that may inform further discoveries and investigations; (iii) association of intraspecific trait variation to geography, ecology, and time which is particularly important for understanding trait variation change; (iv) revision of trait variation within and among species as taxon concepts are revised; and (v) cross-validation of species-level trait data derived from the literature or trait databases found in community resources, such as AmphibiaWeb (http://amphibiaweb.org, accessed 16 September 2016) or Fishbase (http://fishbase.org, accessed 16 September 2016).

The key question is, ‘How can we associate trait data with observations and specimens efficiently?’ Perhaps underappreciated is the vast trove of trait data that is already in digital form, but that has yet to be distributed in ways that make those data discoverable and maximally useful. For well over a century it has been common practice in many natural history collection communities for field collectors to annotate key aspects of the biological state and condition of collected specimens, especially length and mass measurements. These measurements are often written directly onto specimen labels and represent an intriguing potential source of data scattered across thousands of natural history collections globally.

Over the last two decades, an unparalleled opportunity has developed to reassemble this treasure trove of traits into a coherent resource. Currently, biocollections are in an era of rapid digitization (14–18). In many cases, traits are digitized and mobilized into online platforms such as VertNet (15). Information is mobilized through VertNet using Darwin Core (19), which has become a standard for natural history collections data sharing. In Darwin Core there are fields to capture commonly shared attributes such as sex and life stage. Although the fields are well defined, the content of these fields is still quite heterogeneous. For example, in VertNet, more than a 2800 distinct values could be found in the Darwin Core sex field at the time the data for this study were extracted. Nearly all other attribute and trait measurements, however, are relegated to Darwin Core fields that are used by data publishers as containers for comments (*dwc:occurrenceRemarks*, *dwc:organismRemarks* or *dwc:fieldNotes*, where *dwc*: denotes the Darwin Core namespace of terms) or for user-defined attributes-with-values (*dwc:dynamicProperties*). Traits are not often captured in distinct fields in the original data sources, and even when they are, the lack of Darwin Core terms for specific measurements oblige data publishers to aggregate them in these more generic Darwin Core fields. Thus, while trait-related content has always been available to the community via the VertNet portal (http://portal.vertnet.org, accessed 16 September 2016), the ways in which these characteristics have been captured across collections is highly heterogeneous and no mechanism for standardization is currently available. This has rendered discoverability and further use highly challenging.

In this study, we provide a detailed description of a novel process that extracts trait information from specimen records, harmonizes trait descriptions and contents in relation to existing ontologies and best practices, and creates new features in the VertNet portal to enable the discovery of trait content associated with specimen records. We provide statistical summaries of data extracted and an examination of the corpus of trait present in the data beyond what we extracted. In this study we focus on vertebrate records, given the large amount of data already available and the strong efforts already made toward data improvement during publishing (20). Our larger goal is to demonstrate how this overall approach and bioinformatics toolkit can be deployed broadly in support of ongoing major initiatives to digitize all biocollections (14, 21–24).

## Materials and Methods

### Data source—the VertNet portal

VertNet is a well-known and highly-used community-oriented biodiversity data publishing organization (15), which aggregates vertebrate data published by natural history collections from all over the world using the Darwin Core standard. VertNet provides a range of publishing services, access to data via a web portal, and Application programming interfaces (APIs) on a cloud-based platform for data harvesting and aggregation. Data that are accessible via VertNet include specimens and observations associated with taxonomic as well as geographical and geological context. Records are published as Darwin Core Archive files (Remsen et al., 2010) using the Integrated Publishing Toolkit (IPT; Robertson et al., 2014; http://www.gbif.org/ipt, accessed 16 September 2016), a free and open-source web application for biodiversity data sharing developed by the Global Biodiversity Information Facility (GBIF). The published archives are discoverable on the Internet, and in many cases, hosted by VertNet on its own IPT instance. Darwin Core Archives are harvested (https://github.com/VertNet/gulo/releases/tag/v2016-09-16) and indexed (https://github.com/VertNet/dwc-indexer/releases/tag/v20 16 -09-16) using Google-based cloud services. A front-end web application (http://portal.vertnet.org, accessed 16 September 2016, code: https://github.com/VertNet/web app/releases/tag/v2016-09-16) provides search functionality.

### Trait assessment, focal trait extraction and definitions

To assess the complexity of shared trait data we compiled a thorough list of traits as found in relevant Darwin Core fields (see below) across the whole corpus of 18 259 640 records (from 215 data sets shared by 91 data publishers) in the VertNet portal as of 28 October 2015. This compilation was derived from a complete copy of the VertNet data store on that date in a Google BigQuery table called, at the time, ‘vertnet_latest’.

**Figure 1.**
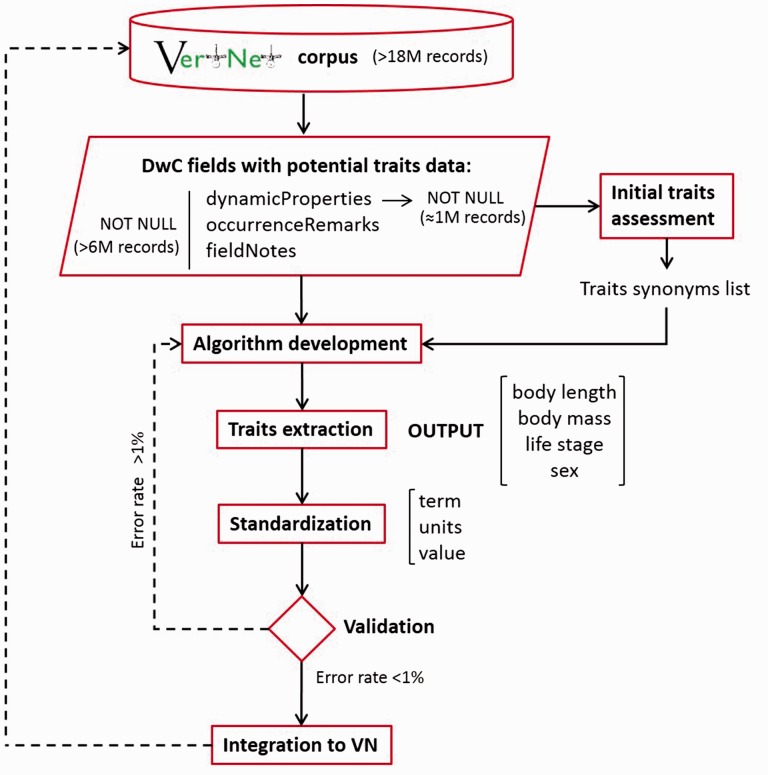
A workflow description for trait extraction from VertNet. The initial extraction of content that bore descriptive trait information yielded more than one million records that served as the basis for initial trait assessments, and then fed into a workflow for finding two traits (body length and mass) and two attributes (sex and life stage).

We used BigQuery to extract records from the vertnet_latest table with content in any of three generic Darwin Core fields where trait data may be located: ‘dynamicProperties’, ‘occurrenceRemarks’ and ‘fieldNotes (Figure 1). Though the Darwin Core MeasurementOrFact class of terms is specifically meant to be used to capture content such as trait values, its use is currently quite limited in biodiversity data publishing. Indeed, not one of the 215 data sets in VertNet included MeasurementOrFact terms at the time this study was done. In the absence of the explicit MeasurementOrFact terms, the field *dynamicProperties* is intended to store trait content, among other things, and is defined as ‘A list of additional measurements, facts, characteristics or assertions about the record’ (http://rs.tdwg.org/dwc/terms/history/index.htm#dynamicProperties-2014-10-23). The use of the Darwin Core standard is not uniform across data sources; thus, trait data can also be found in the fields *occurrenceRemarks* (http://rs.tdwg.org/dwc/terms/ history/index.htm#occurrenceRemarks-2009-04-24) and *field Notes* (http://rs.tdwg.org/dwc/terms/history/index.htm# field Notes-2009-04-24). Therefore, we used all three fields in the analysis. The specific query used to extract the records of interest from BigQuery was:

SELECT * FROM vertnet_latest WHERE dynamicProperties IS NOT NULL OR

occurenceremarks IS NOT NULL OR fieldnotes IS NOT NULL

Our search returned 6 008 472 records having content in at least one of these three Darwin Core fields. This data set (25) became the basis for the rest of the research presented here. Next, we compiled a full list of terms and synonyms from this data set, with special focus on content from *dynamicProperties*, by manually inspecting the 944 059 distinct values in that field. The contents provide a view on data reported in vertebrate biocollections. The set of extracted synonyms provides a coherent basis for further work with this corpus of data. Although not exhaustive, the list is representative of trait descriptions contained within many biocollections. We assembled these synonyms as trait descriptors into broad categories loosely associated with broad classes of body regions, including: head, neck, appendage, tail and trunk, along with body part complexes (e.g. head + body), whole organism characteristics (e.g. total body weight) and non-organismal information (e.g. nest characteristics). We then sorted descriptors into those that were measured as continuous (e.g. tarsus length) or as meristic counts (e.g. clutch size) and those that were qualitative (e.g. color). Finally, we counted how many different descriptors and synonyms were found in each of these broad categories.

In order to gather trait content likely to be common and of broad value to ecologists and evolutionary biologists, we focused our extraction of trait data explicitly on body mass and body length, along with information on sex and life stage. Body mass and length have been shown to be essential integrator variables widely used in ecological and evolutionary studies (26–28) to represent a relatively simple starting point for trait assembly using informatics tools. Sex and life stage are attributes of individuals that are of critical importance for proper interpretation of all other measurements, and so were deemed essential to include in the extraction.

To assure that we used standard definitions for traits, we annotated the extracted data using the Vertebrate Trait Ontology (VTO) (29) for definitions of body length (http://purl.obolibrary.org/obo/VT_0001256, accessed 16 September 2016) and body mass (http://purl.obolibrary.org/obo/VT_0001259, accessed 16 September 2016), while sex (http://rs.tdwg.org/dwc/terms/history/index.htm#sex-2009 -04-24) and life stage (http://rs.tdwg.org/dwc/terms/history/index.htm#lifeStage-2009-04-24) were referenced to Darwin Core definitions. Hereafter we use *vt:body length*, *vt:body mass*, *dwc:sex* and, *dwc:lifeStage* to refer to the specific community definitions given earlier.

### Extracting trait data

The next step was the extraction of the values for the target traits (body length, body mass, sex and life stage) from the ‘dynamicProperties’, ‘occurrenceRemarks’ and ‘fieldNotes’ fields. Extracting the target content was particularly challenging because there is no consistently followed standard to guide how data are recorded on labels or digitized. For example, there are multiple types of body length measurements, some specific to a particular vertebrate subclade, and a variety of synonyms and abbreviations for these distinct measurement types.

Extraction relied on a multi-step process (Figure 1). First, we determined the categories of traits for which we would extract values. In the broader category of *vt:body length*, we established a set of subcategories that were found in the data: *total length*, *head-*‘body length’, ‘snout-vent length’, ‘standard length’ and ‘fork length’. These subcategories reflect distinct community standards and measurement practices. For example, ‘snout-vent length’ is a term used explicitly for reptiles and amphibians (30). Similarly, ‘standard length’ and ‘fork length’ are measurements specific to fishes (31; Florida Museum of Natural History, Ichthyology Collection, http://www.flmnh.ufl.edu/fish/discover/fish/anatomy/features-measurements, accessed 16 September 2016). *Vt:body mass*, *dwc:sex* and *dwc:lifeStage* did not require subcategorizing. After completing this step of defining the categories of traits and auxillary attributes to extract, we refined the original synonym list generated as described earlier, assembling a list of distinct terms that represent ‘body length’ (https://github.com/rafelafrance/traiter/blob/v0.2/lib/trait_parsers/total_length_parser.py#L253) and ‘body mass’ (https://github.com/rafelafrance/traiter/blob/v0.2/lib/trait_parsers/body_mass_parser.py#L211) for use in the subsequent step. The categories ‘sex’ and ‘life stage’ had more limited numbers of variations, and these were incorporated directly into patterns to seek, also in the subsequent step.

The greatest challenge was to develop code to examine each record’s relevant Darwin Core fields and automate the extraction of traits, associated measurements, and units. We developed a regular expression (regex) library, written in Python, to find the substrings corresponding to the trait terms, measurement values, and units related to *vt:body length*, *vt:body mass*, *dwc:sex* and *dwc:lifeStage*. This regex code is available within a Jupyter notebook on GitHub (32https://github.com/rafelafrance/traiter/blob/v0.2/parsers/vertnet_parser.ipynb). We chose to use regex instead of other possible techniques, such as machine learning, because we were concerned that, although the database for training and testing seems large, the variety of synonyms might render too few examples for this alternate approach to be immediately useful. The extraction process required careful ordering of string matching patterns. First, we focused on the most common and explicit strings used to refer to a given trait (e.g. ‘total length:’). Then we searched for rarer and more abbreviated forms (e.g. ‘TL’ or ‘ToL’ for total length) or pattern matches (e.g. recognizing that the first value of ‘235-97-31-25 = 71.9’ represents a total length of 235 mm, and the last value a body mass of 71.9 g). We provide a detailed description of the regex matching in the Jupyter notebook referenced earlier and refer readers there for full details.

To address the challenge of tuning code to limit false positives (FPs) and false negatives (FNs) during extraction we developed unit tests iteratively for all variants of body length and mass descriptions. A set of Python unit test suites was developed using Python’s unittest library. Each test suite is a Python class and every test is a method in that class. We ran the tests from the command line using the default test loader and test runner from the unittest library. These test cases were checked (see the section ‘Validating the extracted content’, below) to assure that intended results were achieved, specifically to avoid new FPs, as new expression extractions were developed and test cases added. This process is also fully documented in the Jupyter notebook. This type of unit testing, however, could not cover all of the possible permutations found in the full data set and required careful vetting and editing discussed in detail in the section ‘Validating the extracted content’, below. The final output from the extractions was a quadruplet for each trait of interest: (i) the original Darwin Core field from which the observed trait was extracted, (ii) the standardized trait type, (iii) the value of the trait and (iv) the units, if reported.

### Standardizing and normalizing contents

Even after successful extraction, trait types, values, and units required additional standardization prior to integration into VertNet (Figure 1). These standardization steps produced what we call ‘harmonized data’. We used the synonym tables we built to generate standard trait types from verbatim extracted keys. For body length, there were five possible standard subcategory types as discussed earlier (e.g. ‘Total length, snout-vent length’). We applied the same approach to body mass, but only had a single valid type, *vt:body mass*, to which all synonyms were translated. To standardize the values for body length and body mass we converted all values to millimeters for body length and to grams for body mass, since these are the units used typically. Still, many records do bear Imperial units such as ‘lbs’ or metric units such as ‘kg’ for larger specimens. In these cases, we converted the reported values to standard units (e.g. 20 kg became 20 000 g). For cases in which units were reported, this was a trivial conversion. Unfortunately, units were not always reported. In some cases, such as common shorthand reporting, units, while not explicit, can still be strongly inferred, as they follow community practices. In other cases, we could not presume to know the units because the values were not recorded using a standardized syntax. In those worst cases, we assumed units were millimeters and grams, but noted in final outputs that those measurement units were inferred rather than reported. For sex and life stage trait data, we did not apply any *a posteriori* standardization to the data we extracted.

### Validating the extracted content

Regular expressions were developed iteratively, with multiple validation steps to test efficiency and to correct for errors in the output. In each validation step, random sets of 500 records (obtained by adding a column to the full set of records, populating it with random numbers, sorting that column, and taking the first 500 records in the list) were manually checked for accuracy from the post-processed records. Accuracy rates were determined by counting the FP (i.e. the information that was not in the data as published for a given trait or that clearly did not represent meaningful trait values, but was extracted by our code) and FN (i.e. the information that was given in the published data, but not extracted by our code) results. We made a distinction between these errors and situations in which there is no explicit string to signify what is being represented (e.g. ‘age’ or ‘total length’), but the values in the field (‘juvenile’ or ‘comments:158 mm’), imply a trait. For completeness, these types of issues were compiled but not included in error calculation.

After a validation step was completed, the code was amended to account for the issues encountered and the process was repeated with the goal of reducing relatively high frequency omission and commission errors and to achieve error rates below 1% for each of the four extracted traits (‘body length’, ‘body mass’, ‘sex’ and ‘life stage’, Figure 1). The presumed final validation set was double-checked against 2,000 random records, representing 8000 individual extractions, and performed independently by the two first co-authors. In order to determine the performance of the code, we calculated true positive (TPR) and true negative (TNR) rates utilizing the Matthews correlation index (MCC) (33, 34). Finally, for the ‘sex’ and ‘life stage’ data extracted, we performed a check to determine how many of those extracted values were also found in the analogous Darwin Core fields, *dwc:sex* and *dwc:lifeStage*. This provided information on how much new data, not already in standardized fields, was extracted.

### Trait data in perspective

In order to understand how trait data are represented across different occurrence records, after the extraction of traits was done, we added those data to the original records and analysed the incidence of traits along taxonomic clades. For all records that had trait data, we first assigned a clade (‘Fish’, Amphibia, Reptilia, Aves and Mammalia) based on the content of the *dwc:class* field. In the clade *Fish* we included: Actinopterygii, Cephalaspidomorphi, Chondrichthyes, Elasmobranchii, Holocephali, Leptocardii, Myxini, Petromyzonti and Sarcopterygii. Records that did not have a reported clade were assigned one according to the metadata of the collection to which the record belonged (e.g. a record in an ornithology collection was assigned to Aves). Although few, there were some records to which no clade could be assigned and were labeled as ‘Unknown’. Once clades were assigned, we determined the vertebrate species names in each clade by one of the following criteria: (i) concatenation of the *dwc:genus* and *dwc:specificEpithet* fields; or, (ii) the first two words in the *dwc:scientificName* field. For length and body mass traits, we determined the number of species that had extracted trait data in three categories—at least 1 record, >10 records and >100 records.

In order to give some perspective on the utility of trait data, we provide an example that demonstrates using aggregated data in outlier detection. Specifically, we have provided an illustration of outlier identification for a particular rodent species (*Tamias minimus*), using data from VertNet extracted with Traiter (Supplementary Materials S1, Supplemental Figure S3).

### Bringing trait data back into the VertNet portal

The next step was to repopulate the VertNet portal with the extracted and standardized data so that they can be easily discovered and used. The records with the extracted traits were archived on the CyVerse Data Commons (25). The extracted trait data have also begun to be included in the VertNet snapshots, which are periodic taxonomic subsets of VertNet records, and are also available on CyVerse (35–39). To extract trait information from the original data, we developed a Python-based application (‘Traiter’ hereafter; https://github.com/rafelafrance/traiter/releases/tag/v0.2), which operates on each row of extracted trait information and provides the following output (and data types): hasLength (boolean); hasMass (boolean); hasSex (boolean); hasLifeStage (boolean); lengthInMM (numeric); massInG (numeric); wereLengthUnitsInferred (boolean); wereMassUnitsInferred (boolean); derivedSex (string—the value from *dwc:sex* if it is not empty, else sex extracted by Traiter); and, derivedLifeStage (string—the value from *dwc:lifeStage* if it is not empty, else lifeStage extracted by Traiter).

In order to include harmonized trait contents in VertNet, we created a fork of the original Traiter code and used it as the basis for a data processing suite (https://github.com/VertNet/post-harvest-processor/releases/tag/v2016-09-16). This suite prepares data harvested from IPTs for indexing, for periodic snapshots, and for the VertNet portal. Specifically, the post-harvest processor creates csv files with fields populated exactly as they will be needed for indexing in the VertNet data store. Each of the new fields is generated using the adopted Traiter code in the post-harvest processor and becomes part of those csv files that are added to the index, which in turn makes them directly searchable as part of each VertNet record. Records with length ranges (e.g. ‘standard length: 20–35 mm’) in the verbatim original are given a value for ‘lengthtype’ from one of five types of length range (total length range, standard length range, snout-vent length range, head-body length range or fork length range) and a value of True for ‘hasLength’. Range values remain in the field in the record from which they were discovered, but the values are not indexed or searchable, nor are they currently available in their extracted form in the Darwin Core archives from the data publishers. Though a range might signify an uncertainty in the measurements, most range values are from specimen lots with more than one individual per lot, as is common especially for fish, reptile and amphibian collections. Since we do not know the measurements for individual organisms in these cases, and because searching for values falling within ranges can complicate both user interfaces and results received from searches, we decided not to index these types of length values. The VertNet portal and APIs were redesigned to support searches not only using the fields in the original records, but also using the trait values the newly added filters such as ‘hasMass’. Screenshots of the new outputs for specimen records and search functions are shown in Supplementary Figures S1 and S2.

## Results

### Current state of trait data in VertNet

**Figure 2.**
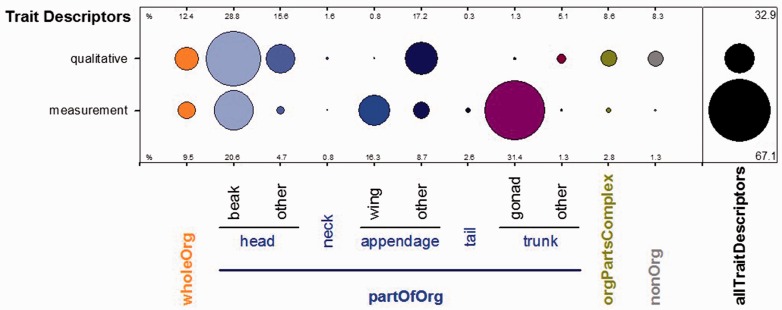
Categorization and relative prevalence of trait descriptors found in the ‘dynamicProperties’ field. Bubbles sizes are proportional to the number of distinct descriptors found for each body region and qualitative or measurement category. Percentages for allTraitDescriptors sum up vertically to 100%, while the overall percentages of qualitative and measurement each sum up horizontally to 100% independent of the allTraitsDescriptors percentages. wholeOrg: terms referring to whole organism characteristics (e.g. total body weight); partOfOrg: terms referring to parts of an organism (e.g. hind foot); orgPartsComplex: terms referring to organism body part complexes (e.g. head + body); nonOrg: non-organismal information (e.g. nest characteristics).

Our analysis of the verbatim content of the ‘dynamicProperties field’ yielded 1,078 distinct organism trait descriptors (738 measurement-related and 340 qualitative available). Here a trait descriptor is an observed quantity or quality expressed as a text string in a particular way. We summarized the overall distribution of trait descriptors in a bubble-gram (Figure 2) by compiling them into categories reflecting body regions. In the figure, bubble size represents the proportion of distinct verbatim descriptors associated with a particular body region. For example, ‘beak’ had the greatest number of overall qualitative trait descriptors, while ‘gonad’ had the greatest number of measurement descriptors.

### Traits extraction and algorithm performance

The extraction code achieved an error rate well below 1%. In particular, we found a total of 62 errors (Table 1: FN + FP) from a check of 2000 records. ‘Sex’ accounted for half of the FPs, which, in almost all cases, was due to parsing verbose information about the sex of more than one individual (not the actual specimen). Conversely, 53.13% of the FNs corresponded to ‘life stage’ and were due to the data presented only as values without a key (e.g. ‘adult’ as opposed to ‘life stage: adult’) and were not recognized by the code. MCCs show that trait extraction performance is excellent (Table 1).

**Table 1.** Code performance assessment: calculation of TPR and TNR rates and of MCC for 2000 records tested for each trait (length and body mass) and attribute (sex and life stage)

	Trait				
		length	mass	sex	life stage
**Indicator**	TP	271	244	611	240
	FP	7	8	15	0
	TN	1705	1735	1359	1743
	FN	10	5	0	17
	**TPR**	0.964	0.980	1.000	0.934
	**TNR**	0.996	0.995	0.989	1.000
	**MCC**	**0.965**	**0.970**	**0.983**	**0.962**

False positive, FP, code extracted information that was not in the original, false negative; FN, code did not extract information that was in the original; TP, true positive; TN, true negative.

Extraction utilizing vetted code (i.e. code that was reviewed after testing units were performed, see the ‘Extracting trait data’ section in ‘Materials and Methods’) yielded results summarized in Table 2, where numbers of records were adjusted according to error rates found for each trait (see more on error rates below). We were able to extract length values from 875 602 records and mass values from 736 891 records. Both body length and body mass were most often captured in the ‘dynamicProperties’ field (80.67 and 92.43%, respectively). The majority of the records that had body length data included units (70.79%; 29.21% required units to be inferred) and 81.16% of the records that had body mass included units. Life stage and sex data were also extracted from the fields ‘dynamicProperties, occurrenceRemarks and fieldNotes’ and their prevalence was compared against that of the corresponding Darwin Core field (i.e. *dwc:lifeStage and dwc:sex*) (Table 2). In the analysed data set, life stage information was found only in other fields in 22.76% of the records, while sex data were found >42 times more often in the Darwin Core sex field than in any of the other three fields (1.27% of the sex data were captured in other fields only). Furthermore, in 97.3% of the records in which sex was found in other fields, it was also recorded in the *dwc:sex* field (45.51% of total records with sex data). Full details of trait content by publisher and collection are shown in Supplementary Table S2.

**Table 2.** Summary results of trait extraction: number of records bearing trait data, with values adjusted for error rates encountered during the extraction process

From record set used (have content in any of ‘dynamicProperties’, ‘occurrenceRemarks’ or ‘fieldNotes’ fields)					From all VertNet
Trait	Only in corresponding DwC field	Only in other field	In DwC field AND in any other field	Total (in DwC field OR in any other)	Total (in DwC field OR in any other)
**length**		875 602	875 602	875 602	875 602
**mass**		736 891	736 891	736 891	736 891
**Sex**	2 045 523	48 613	1 748 730	3 842 866	7 988 634^a^
**life stage**	1 317 110	501 889	385 946	2 204 944	3 234 057^a^
**all traits**^b^	73 106	23	28 664	108 916	108 916

a in all VN (18M records) there are totals of 7940 totals of 7940records) there are totals of 7alues adjusted for error rates encountered DwC fields.

b all traits: records presenting length and mass measurements and sex and life stage data (the latter either in the corresponding DwC fields OR in any other field).

### Trait data in perspective

**Figure 3.**
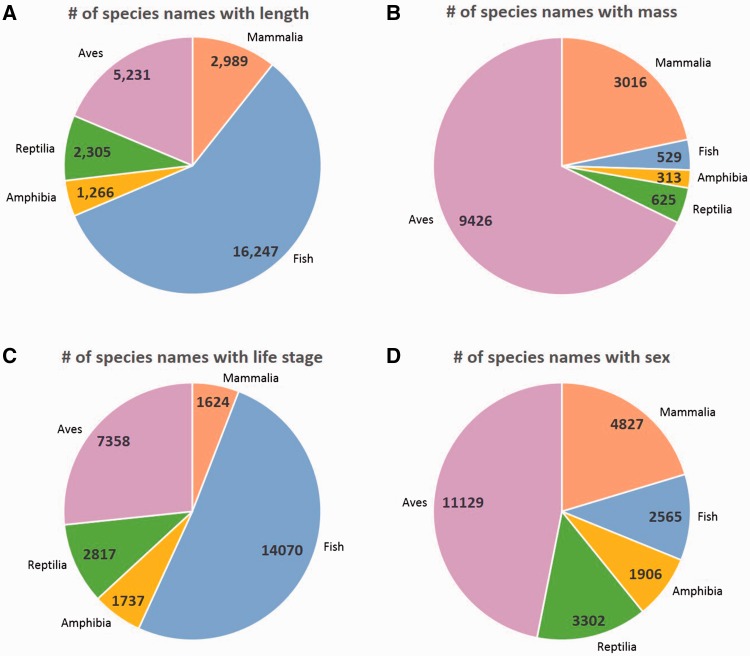
Distribution of species names bearing trait data across the vertebrate taxonomic spectrum. (**A)** Species with a length measurement, (**B**) Species with a mass measurement, (**C**) Species with life stage information, (**D)** Species with sex information. Fish encompasses several classes as described in the ‘Trait data in perspective’ and Materials and Methods’ sections.

We found a considerable number of species that have at least one record with extracted length or mass data. The number of species names for which there were at least 1, >10 and >100 records with length or mass data are shown in Table 3. Several species among Mammalia and Aves were found to have >100 records containing length and mass measurements, while Reptilia and Amphibia records bearing length and mass data are present with less frequency (Table 3). Sex as an attribute was found in at least one record for 23 846 distinct species names, of which 46.7% corresponded to Aves. Life stage was found in at least one record for 27 695 distinct species names, with 50.8% corresponding to Fish. In most cases the number of distinct species names with >10 and >100 records containing these trait data was found to be an order of magnitude higher than those found for length and mass data (data not shown). Of the records that included length measurements, 57.9% corresponded to Fish specimens, making them the most prevalent, followed by Aves specimens (19.3%, Figure 3A). Conversely, Aves was the most represented clade among records bearing body mass measurements (67.8%, Figure 3B). Information for life stage was most often detected for Fish specimens (51%, Figure 3C), while sex was most commonly found in Aves records (46.9%, Figure 3D).

**Table 3.** Species names associated with records containing length or body mass measurements

		Trait					
No. records/sp name		Body length			Body mass		
		>100	>10	at least 1	>100	>10	at least 1
**Class**	Fish^a^	380	3214	16 247	3	14	529
	Amphibia	48	332	1266	21	87	313
	Reptilia	62	507	2305	24	167	625
	Aves	234	1364	5231	706	4050	9426
	Mammalia	417	1161	2989	331	1045	3016
Non_vertebrates		0	0	44	0	0	4
Unknown		0	4	31	0	2	4
**Total**		1141	6582	28 113	1085	5365	13 917

a Fish encompasses several classes as described in the ‘Trait data in perspective’ and ‘Materials and Methods’ sections.

### A new VertNet trait feature

The information extracted using Traiter has been incorporated in the underlying VertNet data store, and the VertNet portal (http://portal.vertnet.org, accessed 16 September 2016) now contains an advanced search mechanism for querying on traits. The advanced search feature, along with results, are shown in Supplementary Figures S1 and S2 for a search where ‘hasMass’ has been selected, and where a search has been done for records with ‘total mass’ between 60 and 70 g. For any record where a trait value was detected, we provide the trait information in the displayed visual output for each record, along with the more typical data contents shown. In all cases, all downloaded data contains the harmonized trait measurements.

## Discussion

### Significant amounts of hidden trait data

Biodiversity collections, in sum, contain an enormous amount of information on the condition, coloration, size and other fundamental characteristics of organisms (21, 40). In this work, we focused on extracting body mass and length measurements from vertebrate occurrence records because these traits are often regarded as integrator variables. We also captured sex and life stage information since these strongly enhance utility of all other trait data extracted. Yet, this only scratches the surface of the data still hidden in specimen records. Figure 2 provides evidence that there are far more trait descriptions for beaks and gonads than there are for body characteristics such as mass and length. There is no question that significant and diverse trait data can be further assembled using smart approaches, akin to other text mining methods for biodiversity data (41). Further, these attribute data need not be measurements of a whole organism or organismal part. Extracting data related to habitats and environment, for instance, could inform research into current and historical microscale distribution of species and populations, help identify sampling gaps, and establish research priorities, especially when done in a way that standardizes habitat descriptions (42). Although our focus was limited to two key traits (body length and body mass) and two common attributes (sex and life stage), we still face many challenges related to harmonization, provisioning of data in the best possible way for end users, and developing new methods and informatics workflows. We elaborate on these issues below.

### Harmonizing the heterogeneous

Although we have demonstrated success in pulling trait data out from VertNet-published records and utilizing tuned regular expressions, our process is not perfect and we acknowledge that improvements and new methods could be employed to further reduce error. Part of the issue is that local data capture process is not homogeneous across data publishers, and the structure of trait data can vary greatly depending on the protocols and tools used, on who performs such capture (experts vs. non experts), and on whether the data have undergone quality controls later in the process. All these factors contribute to the heterogeneity found in trait data, and should be taken into account when developing more powerful tools for trait extraction.

We advocate, in particular, moving beyond simple regular expression matching to methods that utilize more of the syntactic and semantic structure of the content. Such approaches could improve trait extraction, especially when extending to the much larger amount of content beyond simple measures such as body size and mass. We foresee strong value for tools that can quickly and automatically annotate semi-structured text and return matches to terms in core ontologies, similar to what has been produced in the biomedical arena (https://monarchinitiative.org/annotate/text, accessed 16 September 2016; 43). Natural history collections data, however, represent a challenge in that the use of highly abbreviated or special formats is common, and such expressions would likely be missed by tools utilizing natural language, a problem not unique to biodiversity records (44). One possible way to overcome this issue would be to incorporate such abbreviations incrementally into natural language processing tools, therefore augmenting their extraction capacity. Machine learning approaches also hold much promise and are an obvious next step. Finally, we see the value of tools such as Argo (45) for opening up workflows established here and helping with curation.

Another challenge for successfully finding and extracting trait content lies in the heterogeneity in where in the records the information is located. Ideally, we would like to rationalize the content into consistent containers for broadest use of these data, as has been discussed for taxonomic information in (20). A particularly strong example emerges with ‘life stage’ extraction. Darwin Core has a term, *dwc:lifeStage*, which describes a field in which all this content should go. Life stage information, along with sex, is essential for interpretation of any other biological information, thus we made efforts to check if such information might be found as a ‘dynamic property’ rather than in the Darwin Core fields specifically meant for the purpose. To our surprise, we found that over 500 000 records (∼22% of the total number of records we examined) had life stage information in ‘dynamicProperties’, ‘occurrenceRemarks’ or ‘fieldNotes’, and not in *dwc:lifeStage*. This is a significant proportion of content critical for the broadest use of any other trait information that is essentially hidden. Moreover, even when this information is exposed, challenges remain interpreting life stage contents, since the values contained within the dwc:lifeStage field usually do not conform to controlled vocabularies and are often hard to interpret without expertise. Therefore, it is important to highlight a need for better documentation of how to use the Darwin Core standard, and to encourage training that would include examples of use and mapping of original fields to those corresponding to the standard, along with efforts to standardize contents according to community standards. This would foster biodiversity data that is more uniform in structure, ultimately facilitating data discovery. We discuss further issues with fixing such problems for the long term in the following section.

Finally, we note that heterogeneity extends to the actual measurement precision and units of trait data. For example, we found high variation in decimal precision reported, ranging from integers to remarkably over-precise measurements of 10 or 15 decimal places. In homogenizing data, we kept low precision records at the reported values, but uniformly truncated other precision to two decimal places. The field from which data were extracted maintain the values that were reported originally. Such linkage transparency between original and extracted, interpreted data, is particularly critical for measurements, where ancillary data such as when the specimen was measured, and its overall condition (e.g. measures of reproductive state and fat stores), may prove particularly important for interpretation. Finally, harmonization of units is a critical part of the work we performed, especially since units were not uniformly reported in the raw content we extracted. In some cases, one can make almost certain inference that units are in millimeters and grams. For example, certain shorthand reports, such as those used for mammals of the form ‘235-97-31-25 = 71.9’, signify a series of length measurements (here total length, tail length, hind foot length, ear length, weight), and are by custom in millimeters (mm) and grams (g) (46). In other cases, unit inference is uncertain. In these cases, we note that units were inferred without known community practice to guide us, which occurs nearly a quarter of the time.

### Trait assembly in new ways

Our goal with this work was to introduce a new framework for trait data combined with the specimens on which these traits were observed. We did so because we believe that creating public resources such as these, in the context of existing data-sharing platforms, will pay high dividends to the biodiversity community and beyond, as has been seen in other arenas (47). A simple case study focusing on outlier detection is provided in Supplemental Materials S1 and Supplementary Figure S3 showing just one example analysis; extending that case further to examine comparisons with other sources of data is an obvious next step. We note that the goal of this work was not to test a particular question directly, but instead to catalyze the larger community to use these trait data more rapidly and broadly, especially given strong needs for such data in relation to essential biodiversity variables (48). This work sets the stage for making these tools and processes part of the VertNet publication and data quality workflows and, in doing so, points the way for other efforts, as digitized specimen records flood into aggregators such as iDigBio (http://idigbio.org, accessed 16 September 2016; 24) and GBIF (http://gbif.org, accessed 16 September 2016; 49).

Ideally, standardization and tools developed here should be broadly integrated early in data publication workflows rather than post-publication, especially given that it reduces challenges with extraction downstream. However, the best way to proceed with trait publication steps cannot be answered by VertNet data managers alone; instead, it is a larger community conversation that can now begin with data publishers to address their interests and needs. Furthermore, since the Traiter tool we developed works independently of the source of the input text strings, it could be more broadly utilized to extract information that is contained in other sources than in Darwin Core fields, such as extensive information on species traits located in species treatments (50), morphological descriptions (51), or from measures taken from specimen images. In this sense, the potential of these tools and its further application is to be determined by the community needs.

In order to make this and future efforts in trait extraction most useful, annotations should be made that relate this kind of data semantically, ultimately improving its discoverability and allowing cross-linking of data from otherwise unrelated sources (52). In this work, we mapped body length and mass terms to the VTO. Although body mass maps well, body length is more challenging. There are multiple ways to measure lengths of whole organisms, some that are truly *sensu strictu* body length, and some that refer to ‘total length’ and include the head, body, and tail of the organism. We expect that more trait data, related with other organismal entities and with features such as habitat, will also be extracted in the near future, and may face similar challenges with respect to mapping to ontologies. Although some of these traits, such as ‘gonads’, ‘tarsus’ or ‘grassland’, might map well to terms already defined in ontologies such as Uberon (53), VTO, the Environment Ontology (54, 55) etc., others will require working with existing initiatives to add new terms or to further clarify properties in order to enhance integration and discovery (56, 57).

We end by noting an obvious but critical point: All of the trait data we can assemble comes from the hard work of data publishers who have captured and maintained these data and then decided to mobilize them. We expect that there is a large number of length and mass measurements on specimen labels that are not yet digitized, or digitized, but not yet shared publicly. Further, for those specimens lacking any measurement, imaging and measurements from those images provide a powerful impetus for collating new trait measurements. All such observations and measurements ultimately need to be part of true linked and open data framework (58), where trait annotations can be done across the web, and connected more completely with multiple associated data resources, all of which will enable new opportunities in biodiversity science (59).
